# Assessing the burden of rheumatic heart disease among refugee children: a call to action

**DOI:** 10.7189/jogh.06.020305

**Published:** 2016-12

**Authors:** Gabriele Rossi, Vivian Sau Wai Lee

**Affiliations:** 1 Médecins Sans Frontières, Operational Center, Brussels, Belgium; 2 Médecins Sans Frontières Hong Kong, Sai Wan, Hong Kong

In a recent editorial in *The Lancet*, Langlois and colleagues are adamant in advocating for a better and more universal access to healthcare for refugees landing in Europe [1]. The arguments of equity, social justice and cost–effectiveness are clearly explained in their comment.

Among the different suggestions to improve the refugees’ health, the authors invite the policy makers to opt for strategies favoring the “provision of preventive care, including primary and secondary prevention of cardiovascular disease” which “could generate savings for health–care systems by alleviating the burden of stroke, and myocardial infarction”.

Their advice, geared towards the betterment of the refugees’ adult population, needs to be further complemented by a strong call for providing primary and secondary preventive care for acquired cardiac diseases in refugee children.

While acute rheumatic fever (ARF) has essentially vanished from industrialised countries since the latter half of the 20^th^ century, the condition and its major sequel, rheumatic heart disease

(RHD) remains an important public health concern in poor, developing and war–torn countries.

Poverty, inadequate and disrupted primary healthcare systems are major contributors to the persistence and resurgence of ARF/RHD in these countries [2].

The estimate of the global burden of RHD has been recently established at 33 million existing cases, which is higher than the previous conservative 15.6 to 19.6 million cases in 2005 [3]. Emerging echocardiographic data following the seminal work of Marijon and colleagues [4], have contributed in revising these data, suggesting for a higher prevalence of subclinical RHD. In particular, according to a recent meta–analysis by Rothenbuler et al [5], among children aged below 18 years from 37 pooled countries, the prevalence of clinically silent rheumatic heart disease (21.1 per 1000 people, 95% confidence interval CI 14.1–31.4) would be about seven to eight times higher than that of clinically manifest disease (2.7 per 1000 people, 95% CI 1.6–4.4). South Asia and some African countries have very high prevalence data [5].

Although the significance of subclinical RHD is still not clear, follow up studies indicate that most Subclinical Definite RHD (according to World Heart Federation Criteria) [6] and at least a proportion of Borderline RHD, are likely to be true RHD requiring secondary prophylaxis [7].

To tackle the evolution of RHD, specific control programs based on the concurrent development of disease registers along with the consistent delivering of benzathine penicillin G injections, need to be strengthened by a decentralized echocardio–based active case finding activities, comprehensively integrated into the existing primary health care services.

In this sense, the World Heart Federation’s non–communicable disease action plan is calling for a 25% reduction in premature mortality from RHD by the year 2025 (“25 by 25”) [6].

Photo: Médecins Sans Frontières assists Syrian refugees in Roske, at the Hungary-Serbia border. Among the refugees arriving in Europe, half of them are children. Courtesy of Ana Lemos/MSF (2015).

Unfortunately, this cost–effective strategy has not been routinely implemented in low–income and middle–income countries, because of the structural weaknesses of their health systems.

The current enormous refugee crisis in Europe represents a paradigmatic translational shift: the health systems of the wealthy countries are now called to deal, in a substantial way, with the poor people and their diseases.

The Syrian children, suffering from more than five years of displacement, have been forced to live in very dire conditions. Being exposed to the effects of the detrimental combination of seasonal harsh cold weather, overcrowding, poverty and very limited access to healthcare [8], they hold the classic risky profile for the development of ARF and subsequent RHD [9].

With an expected echocardiography–based screening detection rate of around 2%, many migrant children landing in Europe (Syrian, Afghan, Eritrean, Bangladeshi, and many others) could be timely identified and placed under prophylactic treatment for RHD. In 2015, more than 1 million asylum seekers arrived in Europe and half of them were children: the figure of 10 000 diagnosed subclinical and clinical RHD could be anticipated. Many more refugees are expected to land in Europe in 2016.

Pilot studies aimed at proving the validity of these assumptions, while assessing the prevalence of RHD among refugee children, are urgently warranted.

Decentralised diagnostic services for ARF/RHD, framed as routine screening through point–of care technologies (echocardiography and antigen tests for the rapid diagnosis of group A streptococcal pharyngitis) should be then considered at migrant/refugee pediatric community level.

In view of the fact that severe RHD is lethal in the absence of surgical treatment, with a death toll of 275 000/year [10], the same arguments of social justice, equity and cost–effectiveness mentioned by Langlois and colleagues should apply here, especially for the silent and marginalized population of refugee and migrant children, often arriving in Europe unaccompanied and defenseless. Europe has the infrastructures, human and financial resources, and knowledge to act accordingly. It must not turn a blind eye to them.
